# Real-Time Compression for Tactile Internet Data Streams †

**DOI:** 10.3390/s21051924

**Published:** 2021-03-09

**Authors:** Patrick Seeling, Martin Reisslein, Frank H. P. Fitzek

**Affiliations:** 1 Department of Computer Science, Central Michigan University, Mount Pleasant, MI 48859, USA; 2 School of Electrical, Computer, and Energy Engineering, Arizona State University, Tempe, AZ 85287-5706, USA; reisslein@asu.edu; 3 Centre for Tactile Internet with Human-in-the-Loop, Technische Universität Dresden, 01062 Dresden, Germany; frank.fitzek@tu-dresden.de

**Keywords:** tactile internet, real-time compression

## Abstract

The Tactile Internet will require ultra-low latencies for combining machines and humans in systems where humans are in the control loop. Real-time and perceptual coding in these systems commonly require content-specific approaches. We present a generic approach based on deliberately reduced number accuracy and evaluate the trade-off between savings achieved and errors introduced with real-world data for kinesthetic movement and tele-surgery. Our combination of bitplane-level accuracy adaptability with perceptual threshold-based limits allows for great flexibility in broad application scenarios. Combining the attainable savings with the relatively small introduced errors enables the optimal selection of a working point for the method in actual implementations.

## 1. Introduction

Mobile communication network bandwidth has become abundant in recent years, with new use cases emerging predominantly in the Internet of Everything realm. Although initially focused on the Internet of Things, which was dominantly driven by low frequency, low bandwidth sensing and monitoring, use cases have changed dramatically over the past decade. The increasing use of sensing, processing, and actuating control loops across sectors from agriculture over industry to leisure application scenarios has become ever increasing [1,2,3,4,5,6]. More recently, this has resulted in the consideration of these control loops with human operators in the loop, coined as the Tactile Internet (TI) [7,8]. As a result, future communication networks will experience significant challenges to cope with ever-increasing latency demands in general, while the current focus is on industrial scenarios [9,10,11,12,13].

In general, the human–machine cooperation that is at the nexus of several disciplines is gathering an increasing interest. Haptic considerations in the ultra-low-latency context were described in, e.g., [14]. In [15], the authors focus on compression and encoding for tactile (i.e., haptic as well as kinesthetic) information. Some of the original works were described in [16], where the authors provide first forays into perceptually optimized encoding of tactile information. Commonly, perceptual coding approaches are based on the concerns of the Quality of Experience (QoE) or Quality of Interaction (QoI) by exploiting human sensory processing particularities. One such approach is the reliance on *just noticeable differences (JND)*, coined in [17]. The JND principle has been popularly employed in mapping of objectively measured metrics from the Quality of Service (QoS) to derive the human perception-focused QoE [18]. The relationship between QoS and QoE is typically described as a logarithmic one, such as in [19]. The Weber–Fechner law has evolved to a metric allowing a comparison of the impacts of QoS on different types of media, see, e.g., [20].

As human operators are increasingly becoming part of control loops across application scenarios, an increase in bi-directional communications will likely follow [21], which constitutes the Tactile Internet with Human-in-the-Loop (HITL). With different perceptual coding parameters, existing studies in [22,23] exhibit a broader range of packet inter-arrival times than estimated [24]. These could result in significant concerns for these types of application scenarios, which are tightly delay bound around the 1 ms boundary.

The need to deliver round-trip times of 1 ms for sensing, processing, and resultant actuating (inclusive of all networking delays) will put significant strain on existing networks [25]. New approaches are required to reign in latencies, e.g., through processing in the network near the sensing and actuating locations [26,27,28] in conjunction with accelerated processing techniques [29,30,31,32,33]. Current projections for Tactile Internet scenarios [33] allocate the 1 ms delay budget as follows for a generic sensor-to-actuator control loop: 0.1 ms for the embedded sensing processing, 0.1 ms for the wireless and wired transmission and reception processing to the edge computing location, with an additional 0.125 ms budget for propagation delay over a distance up to 25 km. Then, 0.35 ms for the computing processing at the edge computing site, followed by 0.125 ms for propagation, 0.1 ms for the wireless and wired transmission and reception processing, and 0.1 ms for the embedded actuator processing. Thus, the typical delay budget for sensor processing is 0.1 ms, requiring low-complexity sensor data processing—a main design imperative for BINBLISS. Also, specific strategies for long-distance robot control loops are needed, for example, the model-mediated virtual reality remote robot control systems, e.g., the so-called digital twin technology [34,35,36,37,38], can overcome the latency challenges for sensor-to-actuator loops that span long physical distances for automated or non-automated control loops. Although initially, concerns for single data streams can be alleviated by focusing on the relative infinite bandwidth (several bytes of sensed data vs. several gigabytes of network bandwidth), the capacity of networks, especially in dense deployments could become a future bottleneck.

Data compression is generally desirable in sensor networks to alleviate such a bottleneck [39]. The density can stem from dense population or machine deployments, such as in metropolitan areas or in factories, but also be the result of an increase of the sensors/actuators that are placed, e.g., on individual operators (humans or robots) [40]. A common example is, e.g., a tactile glove [41] or similar sensing garments [42,43]. Although the actual underlying sensing/actuating might not be readily modifiable to result in a reduction of latencies, the sending of multiple of those readings could yield compression benefits, if it were possible to perform compression in real time to avoid significant negative impacts on the overall system latency.

This article describes our BINBLISS algorithm that can be executed at endpoints almost without delays by compressing the actually transmitted numbers. We note that a preliminary abridged version of the BINBLISS algorithm has been presented in the conference paper [44]. This journal article substantially extends the prior conference paper presentation with (i) an expanded description of the BINBLISS algorithm in Section 2 with a novel algorithm for incorporating perceptual change into BINBLISS (see Algorithm 1 in Section 2.2), (ii) an expanded compression evaluation with TU Munich kinesthetic traces and JIGSAW traces of robotic surgery in Section 3 (whereas [44] gave only a few compression results for the TUM traces), and (iii) a novel combined performance metric definition with extensive numerical evaluation in Section 4.

We note that this present study focuses on the compression of the tactile Internet data stream; and may be combined with compression of other data stream components, e.g., video streams [45,46,47], and compression of the Internet protocol headers [48]. We furthermore note that in this article, we focus on the theoretical impacts based on the previously described data sets, leaving human subject experimentation for future studies. This article provides the full conceptual description of our approach, including the pseudo-code algorithm, to facilitate these future studies.

The remainder of this manuscript is structured as follows. Section 2.1 describes the overall compression algorithm while Section 2.2 describes the overall evaluation configuration and methods in greater detail, followed by data set descriptions in Section 2.3 and Section 2.4 for kinesthetic codec development and robotic surgery training, respectively. Section 3 provides the performance evaluation results for compression savings and losses before we describe a combined metric in Section 4. We conclude with a discussion and outlook on future works in Section 5.

## 2. Materials and Methods

We commence this section with a brief description of the Binary Indicated Numbers with Bit-Level Integrated Scalability Support (BINBLISS) real-time compression algorithm. We follow with details of the overall configuration and kinesthetic and surgery data sets we employ throughout.

### 2.1. Bit-Level Integrated Scalability Support (BINBLISS)

**Figure 1:**
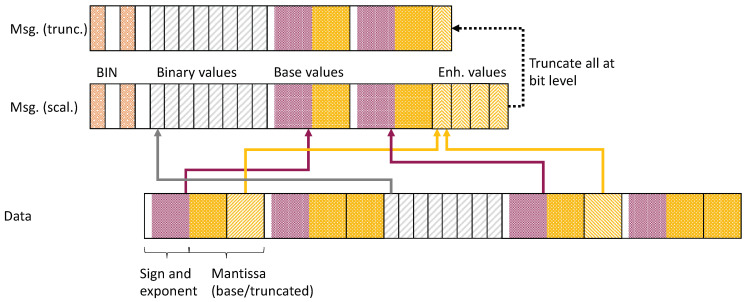
BINBLISS low-latency compression concept: Floating-point values are truncated in granularity, delta-coded with a threshold, and subsequently repackaged by adding binary indicators for the non-changed and modified values. Truncated bits can be added at the message end to enable straight-forward bit-level scalability.

Consider an initially lossless compression approach that employs delta-coding on a per-value basis. A fixed number of values (such as originating from, e.g., a frequently repeated sensor reading) needs to be communicated in well-determined messages. Individual values are always in the same position within a message, which enables a fundamental indicator to signal whether a changed value is contained in a specific message. This binary indicator is prepended to the original message and allows the skipping of those values that are not contained in the message as they are unchanged with respect to the prior message. This approach is lossless, but introduces a small overhead for the new binary header. A single start delay is incurred in form of the very first message composition (in form of, e.g., one sensor reading), after which the delays are only dependent on the processing speed. We provide a visual representation of the packaging process for multiple floating-point values into BINBLISS messages in Figure 1. Other values, such as communicated binary flags, can readily be incorporated as well through simple reordering of a message’s content.

For example, four floating-point values are sent with several binary flags as data in Figure 1. They are re-ordered, and changes evaluated. Here, it is assumed that only the first and third values have changed and are indicated in the new binary header that is followed by the re-allocated binary values.

Additionally, the floating-point values themselves can be modified as well, leading to additional savings through lossy, but speedy compression. BINBLISS enables arbitrary truncation of the number of bits used for the significand or digit part of floating-point values, as in the common IEEE 754 standard [49]. This bit reduction reduces accuracy, but yields additional compression data savings. The removed bits can be appended to the message and be dynamically used or dropped by intermediate or receiving nodes, resulting in a bitplane-level scalability. Figure 1 illustrates this principle by moving the base values (exponent part and truncated significand part) into the main message and the removed bits are appended after all contained values in round-robin fashion, enabling the bitplane-level scalability, which subsequently can be truncated as needed.

Extending the initial consideration for changed values to include perceptual coding approaches, we additionally enable the option of including only values that have changed from the last communicated value by a certain threshold limit. This approach initially performs any value operations, such as truncation of bits to a certain number of significant bits, before comparing the resulting value to the last communicated one. The result is an effective perceptual coding approach, similar to those discussed in, e.g., [15,16,23].

### 2.2. Configuration

We now describe the overall configuration and approach before we explain individual employed data sets separately in greater detail in the following subsections. Commonly, TI data streams exhibit high frequency readings of individually sensed values. One example could be the torque determined in a specific direction at a collaborative robot’s axis to enable safe collaboration. Let the *i*-th of such general values be denoted as $x_{i}$. More specifically, *i* denotes the place of $x_{i}$ in a time-dependent series of values as they are captured. Following the outline in Section 2.1, we retain the general view of i=0,…,I−1, i.e., we generically consider *I* ordered data points (noting that the initial pre-sample would be $x_{-1}$). Employing this ordered list of values, we determine the average and the standard deviation, respectively denoted as μ(x) and σ(x).

An evaluation of modified values with respect to their original counterparts is performed between the original $x_{i}$ and the modified version ${\hat{x}}_{i}$. One of these modifications is the described removal of bits from $x_{i}$ to the internal value $\dot{x_{i}}$. The second change represents the skip of values, i.e., we maintain the previous changed value ${\dot{x}}_{j}$ if the change since the last modified value is below the pre-determined (perceptual) threshold level *l* as in Algorithm 1. (We note that in our evaluations, we always use the first value encountered, i.e., $x_{0}$, effectively assuming that *l* was exceeded.)
**Algorithm 1:** Perceptual change incorporation into BINBLISS.
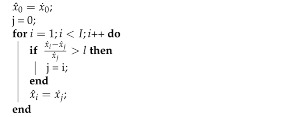


Subsequently, we determine the Mean Squared Error (MSE) as

(1) $$\mathrm{MSE}\left(x,\hat{x}\right)=\frac{1}{I}\sum_{i=0}^{I-1}{\left(x_{i}-{\hat{x}}_{i}\right)}^{2}.$$

Moreover, especially for small values, it might be equally interesting to derive an overview absolute deviation metric. Here, we consider the Median Absolute Error (MedAE), which is less prone to outliers as an additional benefit, as

(2) $$\mathrm{MedAE}\left(x,\hat{x}\right)=\mathrm{median}\left(|x_{0}-{\hat{x}}_{0}|,\ldots ,|x_{I-1}-{\hat{x}}_{I-1}|\right).$$

Oftentimes it is also desired to evaluate relative metrics that can be observed to compare different application scenarios. Let ρ denote the ratio between values as $\rho =\hat{x}/x$. We subsequently consider the relative average and standard deviation as μ(ρ) and σ(ρ), respectively. Other metrics can readily be obtained in a similar fashion.

### 2.3. TU Munich Kinesthetic Traces

We employ the reference traces for codec development from the IEEE P1918.1 Tactile Internet Working Group’s Haptic Codec Task Group. Specifically, these traces were generated as described in greater detail in [50] using a well-defined reference setup. The resultant data set provided by the Technische Universität München (TU Munich, TUM) contains three static and three dynamic interactions, each with 1 ms spaced values that are for force, velocity, and position in each of the three dimensions. We consider the motion vector contents as inputs and the resulting positions as outputs to determine the overall system performance. Each data set covers 25 s of interaction. In our evaluation, we consider the motion vector trace contents x,y,z individually (i.e., as $x_{i}$ inputs as in Section 2.2). Furthermore, we consider the combined origin vector length as input, i.e., we employ $v=\sqrt{x^{2}+y^{2}+z^{2}}$. The individual raw values in the data set are interpreted in our performance evaluation as 32-bit float values. In our performance evaluation, we provide the aggregated outcomes from the individual traces combined.

### 2.4. JIGSAWS Traces

The JIGSAWS data traces are based on data captures from the DaVinci robotic surgery application, described in greater detail in [51]. Specifically, the data set is derived from various training tasks performed and captured at a fixed frequency, based on the manufacturers tool chain. The data set’s individual human subject driven evaluations each consist of 76 kinematic floating-point variables for each data point. These values represent the states and current kinematic properties of the two human operator input interfaces and the surgery robot’s arms as well as the tools attached to them. Each tooltip or effector is characterized further via 3-d coordinates, velocities, and angles. The DaVinci tele-surgery robot features Maxon motors, noting that the highest resolution reported for the company’s encoders is 32,768 counts per turn. (However, it is debatable whether this precision can be achieved jointly with the motor actuation under load, even with micro-stepping.) Raw values in the data set are again interpreted in our performance evaluation of BINBLISS as 32-bit float values.

Incorporating the data set to represent the changes that are caused by BINBLISS between human operator and tooltip/effector requires some additional modifications as follows. Let $x_{i}^{M1},y_{i}^{M1},z_{i}^{M1}$ denote the coordinates of the first human operator manual gripper in the JIGSAWS traces, respectively. Furthermore, let $x_{i}^{P1}\left(x_{i}^{M1}\right),y_{i}^{P1}\left(y_{i}^{M1}\right),z_{i}^{P1}\left(z_{i}^{M1}\right)$ denote the patient-side robot tool tip positions that correspond to the first human operator gripper’s actuation. (We note that we evaluate only the first tool set’s data throughout, as the second set would have similar characteristics.) Dropping the index *i* for readability, we now determine the impact of our BINBLISS approach as follows. We initially determine the factors between the two individual *x* coordinate pairs as

(3) $$x^{M1,P1}=\frac{x^{M1}}{x^{P1}\left(x_{i}^{M1}\right)}.$$

This captures the dynamic fluctuations between manual gripper and effector which are present due to internal DaVinci processing. Let ${\hat{x}}^{M1}$ denote the modified (compressed) value of $x^{M1}$ as in Section 2.2. We subsequently determine the result of compressing the operator’s input value for the robot arm’s output as

(4) $${\hat{x}}^{P1}=\frac{{\hat{x}}^{M1}}{x^{M1,P1}}=\frac{{\hat{x}}^{M1}\cdot x^{P1}\left(x_{i}^{M1}\right)}{x^{M1}}.$$

Other dimensions as well as the origin vector’s length are determined in a similar fashion. Subsequently, all performance evaluations are conducted on the patient side only, as we focus on the resulting impacts of BINBLISS, i.e., we use $x=x^{P1}\left(x^{M1}\right)$ and $\hat{x}={\hat{x}}^{P1}$. We again note that we provide aggregate statistics for the performance evaluation, but group these by activities, namely *Knot* tying, *Needle* passing, and *Suture* creation.

## 3. Compression Results

In this section, we discuss the compression results with a focus on the BINBLISS outcomes for introduced errors with different perceptual and non-perceptual coding configurations as well as for the attainable savings.

### 3.1. TU Munich Kinesthetic Traces

**Figure 2:**
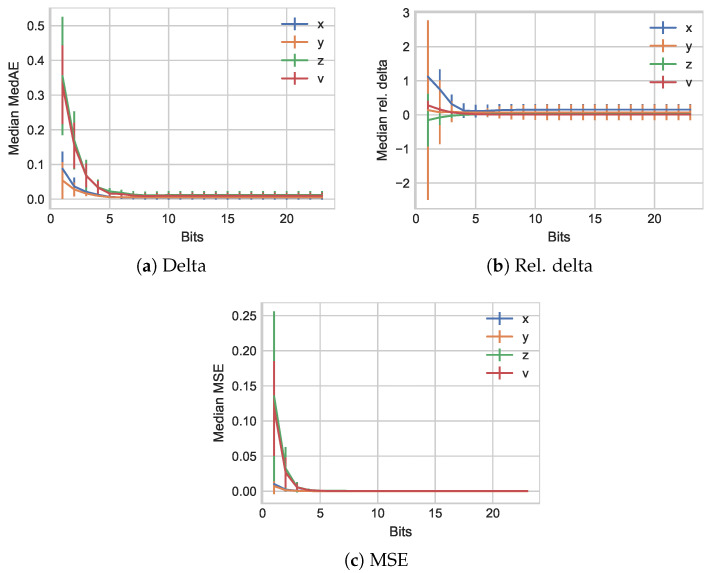
Median results for the *TUM Kinesthetic* data set, evaluated with forced repeats every measurement and no limit (effect of bit reduction only).

We initially investigate the basic effects of the binary compression scheme alone, i.e., we employ a forced repeat on every individual value, independent of content changes. Figure 2 illustrates the resulting medians and standard deviations of outcomes for the TUM data set for absolute deltas, relative deltas, and the MSE, respectively.

We observe a significant drop in the delta as the number of mantissa/significand bits increases, with more than 9 bits not yielding any additional improvements. In turn, we can derive that the overall granularity of measurements could likely be well-captured with 16-bit floats. The exponential decrease of errors, further separated into the three dimensions x,y,z as well as the combined origin vector length *v*. We note that the dominant deviation is for the *z*-axis, which is almost the same as the resulting *v*. Once the overall threshold of about 4–5 bits is passed, the changes become minor. However, one must put these changes into perspective of the underlying base values. The relative delta showcases that initially, very high deviations occur, which rapidly fade. The relative displacement of *v* as result of the compression indicates that the combined effect of the compression might be less noticeable than the absolute values indicate, especially considering for more than 3 bits. A view of the MSE results additionally showcases the impacts we noticed when considering the absolute delta values, though more pronounced. Again, the median MSE drops sharply after 3 bits.

**Figure 3:**
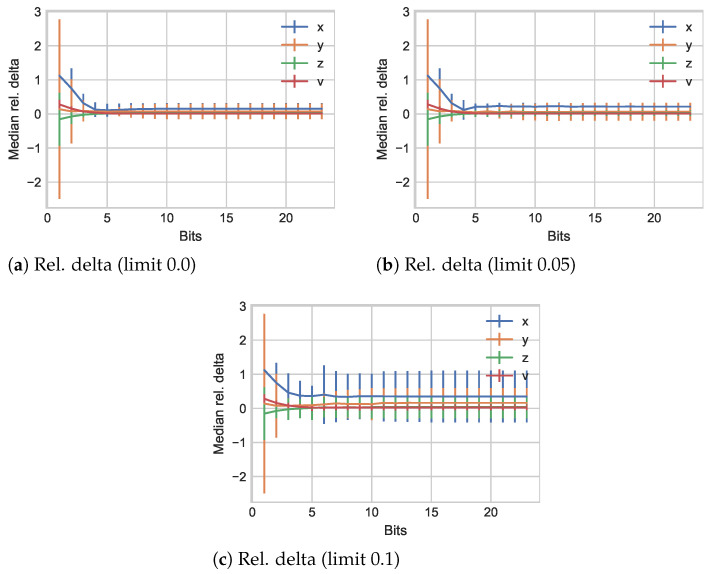
Median relative delta results for the *TUM Kinesthetic* data set with different limits as change thresholds for perceptual coding.

Next, we shift our evaluation to enable a foundational change-based skipping of values. Should either *x*, *y*, or *z* value not be changed, it will be dropped and indicated as described in Section 2.1. The median results for different relative limits *l* are illustrated in Figure 3.

We note that the relative limit of l=0.0 represents the sending of any changed value, while the remaining two examples can be regarded as perceptual coding approaches that limit required changes to 5% and 10%, respectively. For the regular delta-coding paired with the bit-level compression of BINBLISS in Figure 3a, we observe a similar behavior than for the forced updates, with rapidly decreasing impacts. However, some small changes remain due to employed number transformations for statistics overall (we note that we add an infinitesimally small number to avoid divisions by zero). This behavior is to be expected, as small changes are captured in the same fashion that all changes are captured when sending every value independent of changes. Increasing the threshold to 5%, we observe that predominantly the *x* dimension values result is shifted and exhibits a higher relative error level. A similar behavior is visible when considering a further shift of *l* to 10%. There is, however, little impact on the median combined metric *v* for both cases. Only in the last case does the *y* value exhibit a visual increase in the median relative error.

**Figure 4:**
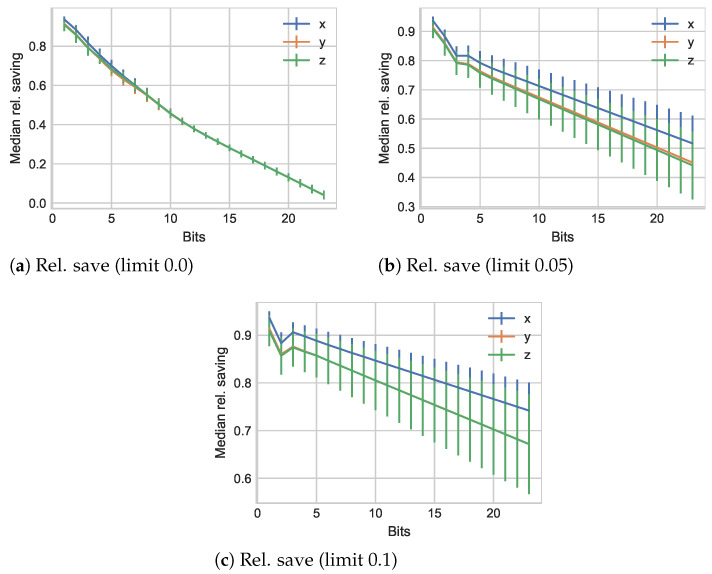
Relative savings results for the *TUM Kinesthetic* dataset with different limits as change thresholds for perceptual coding.

We now shift the view from the introduced errors to the attainable savings for these three cases in Figure 4.

We initially observe significant changes for all three values around 85% as maximum when using only one bit for the mantissa. Although there is a minor variation in between the three axes’ values, overall, they remain close. Increasing the number of bits almost linearly decreases the attained savings with values beyond 9 bits identical for all three dimensions. As the change limit is increased to 5%, we notice a significant separation of the *x* and y,z value results. Specifically, we observe that the relative savings for y,z values decrease faster as more bits are added. A slight plateau forms between 3 and 4 mantissa bits. We also note that the highest overall relative savings level for *x* values is now close to 95%, while y,z values trail by a few percent. One of the reasons here could be the actual source of the data, with less dynamic data in y,z directions and more on the *x* axis. Increasing the change limit threshold for perceptual coding considerations further, we notice an increase of the prior observations. Noteworthy here is the slight dip at 2 mantissa bits, followed by a rise and subsequent linear trend as before. The savings shifts and plateaus can be explained by the different captures of difference levels in the available bit levels.

### 3.2. JIGSAW

**Figure 5:**
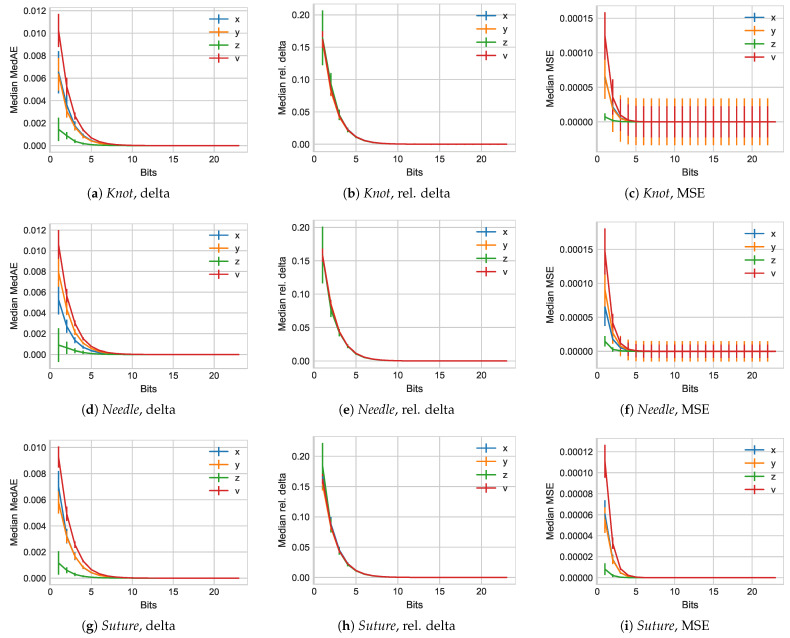
Median error results for all subject experiments in the *Knot*, *Needle*, and *Suture* groups from the *JIGSAW* data set.

The JIGSAWS data set evaluation considers the three separate user interactions of *Knot* tying, *Needle* passing, and *Suture* creation. Figure 5 illustrates the results for these three different application scenarios. We again begin with a view on the compression without delta-coding first.

We initially observe that all three exhibit trends similar to those we observe for the *TUM* data set as well, with slight variations. Here, for all three data sets, the median vector displacement length is highest, with commonly the *z* axis value being lowest. One of the reasons here might well be that the overall movements are higher in their dynamics, but the actual displacement error on the robot’s patient side is lower. Moving on to the relative delta, we notice an upper limit of about 20% for all, which exponential drops similar to prior evaluations. For the *JIGSAWS* data set, we notice a significantly tighter range of all 4 evaluated values than we observe for the *TUM* data set. As before, the MSE reflects the overall observations from the median actual delta.

**Figure 6:**
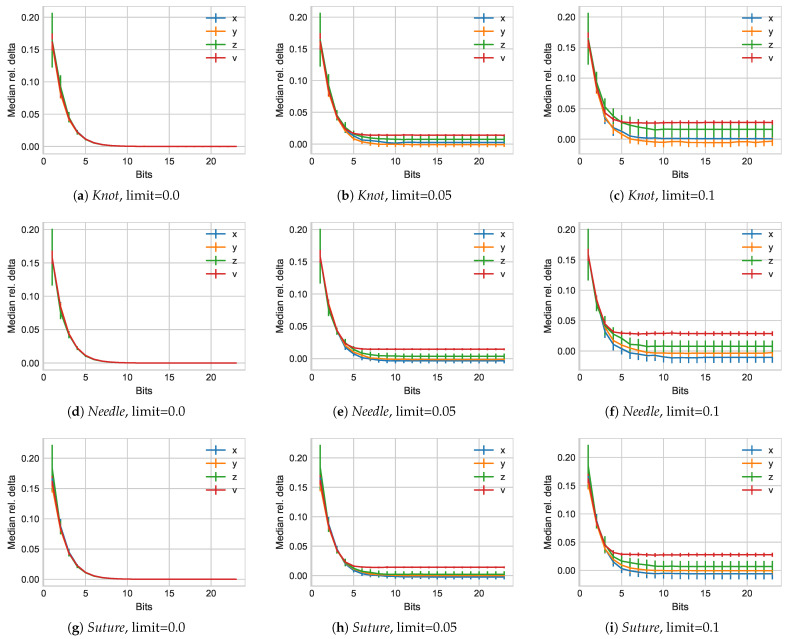
Relative delta results for all subject experiments in the *Knot*, *Needle*, and *Suture* groups from the *JIGSAW* data set.

Next, we include the delta-coding approach of the BINBLISS method, again with 0%, 5%, and 10% relative change between values as threshold limits. Figure 6 illustrates the median result outcomes for the relative errors.

We initially observe the same behavior as before with a limit of l=0 resulting in almost the same outcomes that one would obtain if delta-coding were disabled. As a limit of 5% changes enables perceptual coding, we observe a separation of the four different values, with *v* exhibiting the highest relative delta overall. Although for very low numbers of bits employed all exhibit median relative deltas around 17.5%, this quickly diminishes to about 2.5% when 5 bits are employed. Overall, this also seems to provide an overall threshold for the displacement vector to remain stable while the remaining x,y,z components continue to drop slightly lower until reaching 9 bits. As before, increasing the perceptual limits further results in additional error increases, albeit at a lower level.

**Figure 7:**
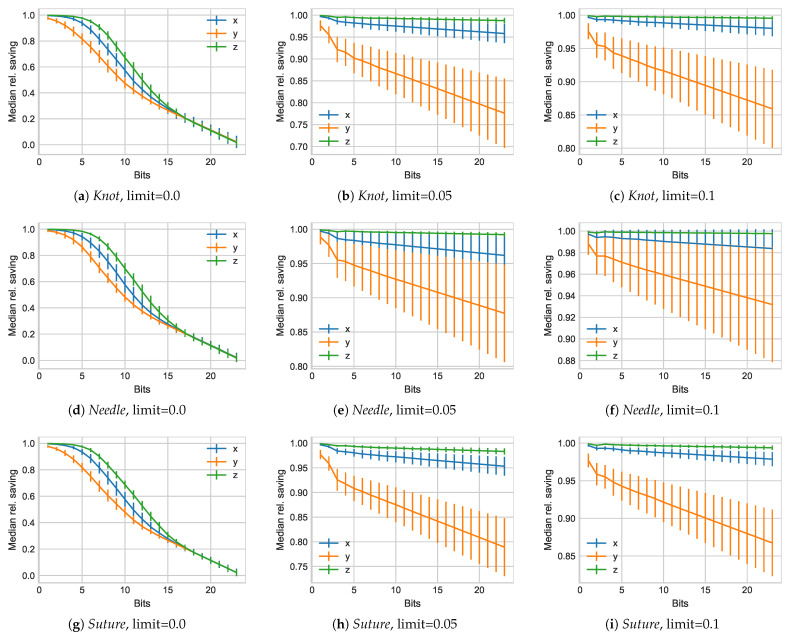
Relative saving results for all subject experiments in the *Knot*, *Needle*, and *Suture* groups from the *JIGSAW* data set.

We now contrast these introduced errors with the attained savings in Figure 7.

We initially observe that a limit of l=0.0 results in a hysteresis resembling spread between the x,y,z values, with *x* providing the highest savings, followed by *z* and *y*, respectively. A removal of all but one bit results in almost 100% savings, as the granularity of changes is no longer captured at all. As the numbers of bits increase, the savings slowly diminish, but even at 9 bits, removing subsequent identical values results in more than 50% savings across application scenarios. A particular reason for this can be seen in the large number of values per data set entry, which include longer periods of inactivity. Once perceptual coding is enabled by allowing a limit threshold of 5%, variability, as captured by the standard deviation across the various human subject traces, greatly increases. The overall trend shapes now more closely resemble those we obtained for the *TUM* data set. We additionally observe significant lower savings for the *y* component than for x,z, which can be attributed to the nature of the source data exhibiting significance lower changes in the x,z dimensions.

## 4. Combined Performance Metric

**Figure 8:**
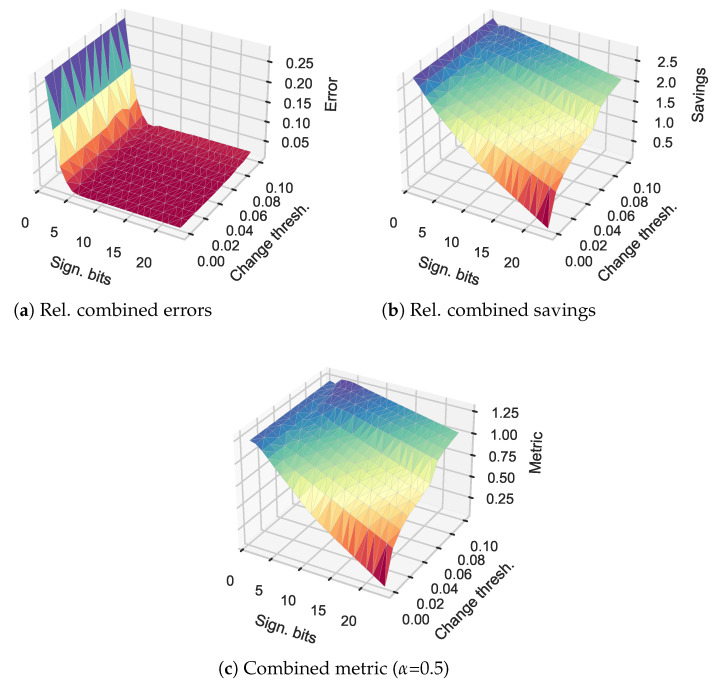
Relative errors, savings, and combined metric results for the *TUM Kinesthetic* dataset with different bit limits and change thresholds for perceptual coding.

In this section, we provide an initial performance metric that combines the relative savings as well as the relative errors introduced to determine an ideal operational point. We provide an overview of the median relative combined errors, savings, and a combined metric as result of the interplay of bits and perceptual change threshold in Figure 8 for the TUM traces. We provide this overview for the combined error $v_{e}rr$ and the individual combined savings $x_{save},y_{save},z_{save}$. We finally combine the two in a simple metric as

(5) $$V_{c}=-\alpha *v_{err}+\left(1-\alpha \right)*\left(x_{save}+y_{save}+z_{save}\right),$$

which allows the weighing of errors and savings differently. In Figure 8, we employ α=0.5 for a balanced view.

We initially note that the relative overall error is primarily dominated by the number of bits employed, and only to a smaller degree by the coding threshold. This is in line with the prior evaluations in Section 3.1. Furthermore, we notice a waterfall-type drop-off for the combined savings. Here, the impact of the perceptual coding threshold is not negligible and contributes significantly to the savings when paired with a reduction in bits. Ultimately, this illustrates the combination of savings in bits per message paired with message content reductions. We notice that the combined result resembles more closely the savings rather than the combined errors, with a maximum $V_{c}$ at 4 bits and a perceptual delta limit of 0.1 (which represents the upper limit employed).

**Figure 9:**
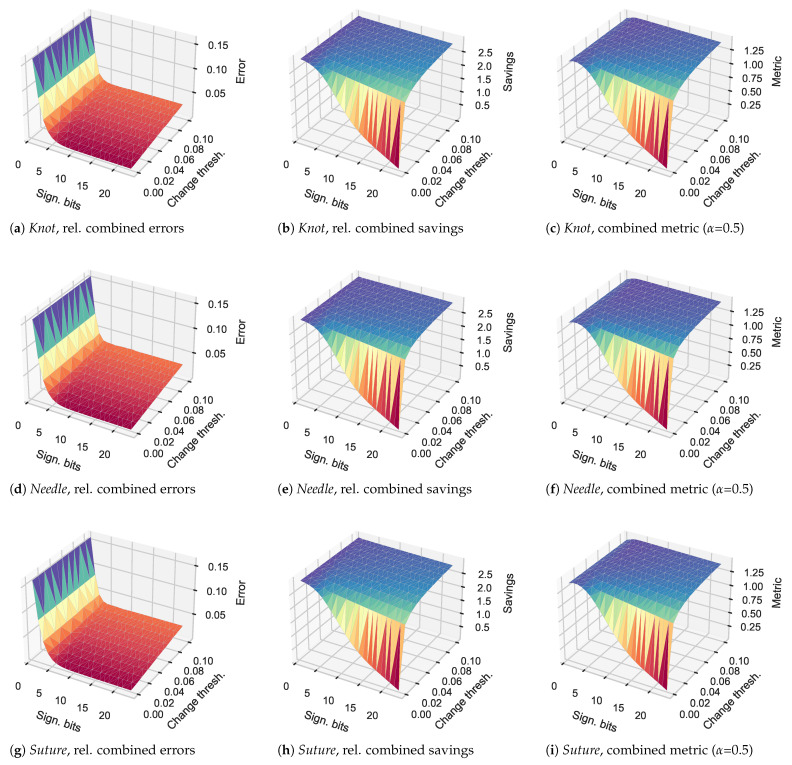
Relative errors, savings, and combined metric results for the three different *JIGSAWS* datasets with different bit limits and change thresholds for perceptual coding.

We perform the evaluation for the *JIGSAWS* data set as well, with results illustrated in Figure 9.

We observe that overall, the results mimic those we observed for the *TUM* data set, but with a more pronounced drop in the combined savings. Each of these scenarios exhibit their maximum at 4 bits and highest evaluated perceptual delta-coding limit of 10%.

The presented results are based on our generic BINBLISS approach and its evaluation without impacts on task performance or human perception. Determining these impacts requires an initial evaluation with human subjects and Institutional Review Board oversight, which is out of the scope of the present study. The BINBLISS approach specified in this article is highly flexible to facilitate a wide range of future research studies and implementation experiments. In particular, the presented BINBLISS design allows implementations to readily adjust the combination of different compression factors and impacts on granularity in specific evaluation scenarios with human subjects.

## 5. Conclusions

This article examined the trade-off between time-sensitive compression based on foundational floating-point number accuracy reduction and the resulting errors for real-life scenarios common to the Tactile Internet: kinesthetic movement and tele-surgery. A significant reduction in data can be obtained through a reduction in the number of bits, with only minor impacts on the resulting error, a benefit for this real-time approach. Similarly, by an introduced delta threshold, further reductions can be achieved that take perceptual considerations into accounts and result in only minor increases in the introduced errors. Combining the two individual outcomes, we derive a main view on the saving/error trade-off, which can be adjusted as needed to determine favorable operational points.

The present study can serve as basis for several important future research directions. One direction is to refine the presented approach through detailed timing measurements in testbed implementations to achieve further data reductions under real-time constraints. A first prototypical (non-optimized) implementation employing Python on an i5 CPU incurred around 0.3 ms of added sending delay on general-purpose computing hardware. We believe that implementation optimizations can substantially reduce this delay. Additional optimizations, including dynamic configuration options for sender and receiver, provide interesting avenues for future research. Based on the BINBLISS approach specified in this article, future research and development efforts should create testbeds to enable more fine-grained evaluation measurements. Such testbeds should also enable human subject experimentation to determine the impacts of our approach on actual task completion performance as well as perceptual impacts, e.g., through experience sampling [52].

Another direction is to explore combinations of the source compression of the tactile Internet data stream with channel coding to make the tactile data stream transmissions robust against network impairments, e.g., wireless network errors. One potential approach is to explore flexible low-delay network coding that may interleave the tactile data stream with related data streams [53,54,55,56]. A broad future research direction is to explore how tactile data stream compression can cooperate with distributed computing, e.g., through multi-access edge computing or fog computing [57,58,59,60], of the tactile signals close to the sensing and actuation sites to achieve ultra-short round-trip computing for tactile applications.

## Data Availability

All data related to this study is explicitly plotted in the figures in this article.
